# Supplementary material for: Molecular Plasmonic Silver Forests for the Photocatalytic-Driven Sensing Platforms
Source: Nanomaterials (Basel). 2023 Mar 2;13(5):923. doi: 10.3390/nano13050923 (PMC10005408; doi:10.3390/nano13050923)
Supplement: Supplementary file 1 [file nanomaterials-13-00923-s001.zip › nanomaterials-2167991-supplementary.pdf]

Supporting Information

Molecular plasmonic silver forests for the photocatalytic-driven sensing platforms

Maxim Fatkullin[1], Raul D. Rodriguez[1], Ilia Petrov[1], Nelson E. Villa[1], Anna Lipovka[1], Maria Gridina[1], Gennadiy Murastov[2], Anna Chernova[1], Evgenii Plotnikov[1], Andrey Averkiev[1], Dmitry Cheshev[1], Oleg Semyonov[1], Fedor Gubarev[1], Konstantin Brazovskiy[1], Wenbo Sheng[3], Ihsan Amin[4], Jianxi Liu[5], Xin Jia[6],** Evgeniya Sheremet[1]*

[1]Tomsk Polytechnic University, 30 Lenin Ave, 634050 Tomsk, Russia

[2]State Key Laboratory of Solid Lubrication，Lanzhou Institute of Chemical Physics，Chinese Academy of Sciences，Lanzhou 730000，China

[3]Van't Hoff Institute of Molecular Science, University of Amsterdam, Science Park 904, 1098XH, Amsterdam, The Netherlands

[4]School of Materials Science and Engineering, Northwestern Polytechnical University, Shaanxi 710072, China

[5]Shihezi University, Shihezi 832003, China

*raul@tpu.ru

**jiaxin@shzu.edu.cn

*To whom correspondence should be addressed

Note S1. Laser-speckle visualization

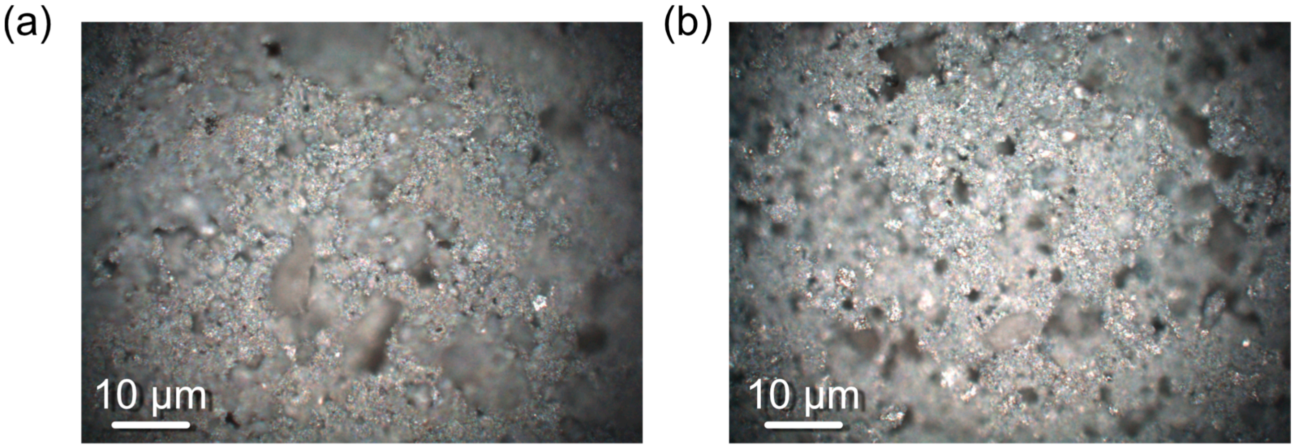

Figure S1 Optical images of (a) Ag NPs film on PET before laser processing, and (b) AgLIMPc.

Laser treatment in a single spot was done in a single spot

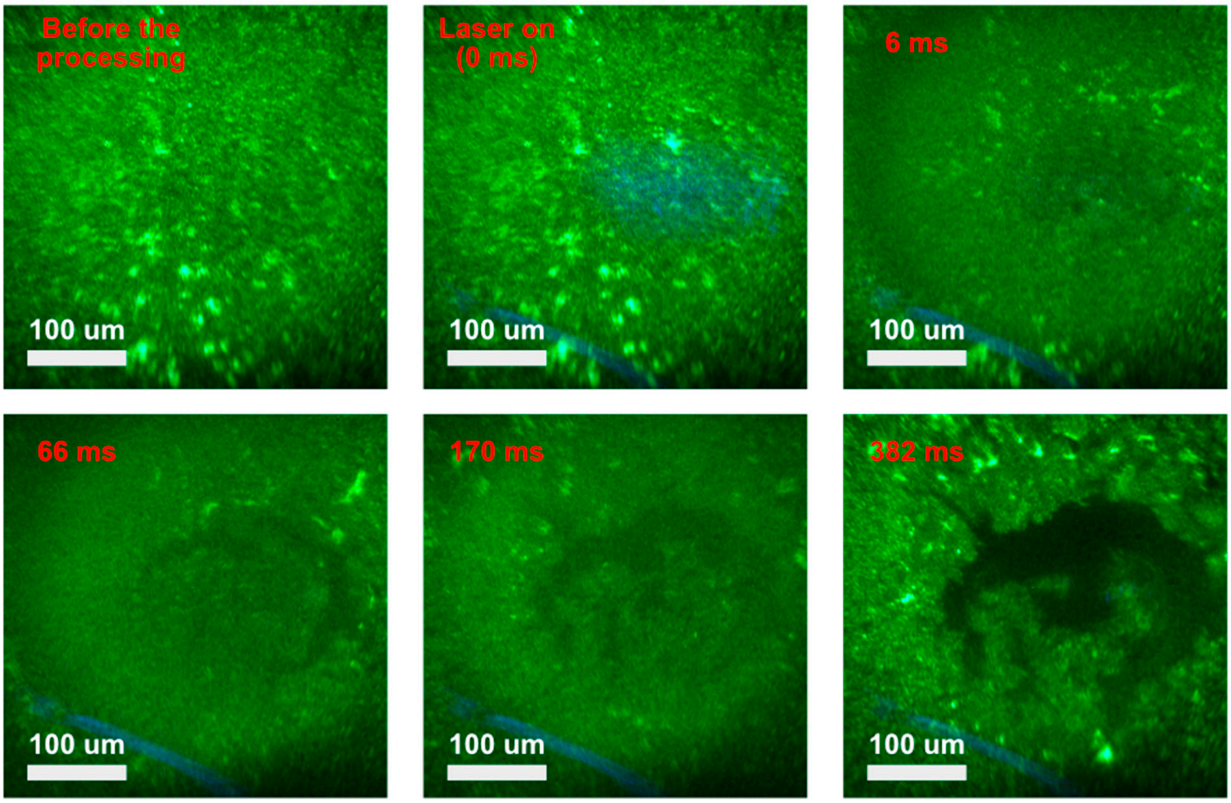

Figure S2.Time-resolved high-speed camera visualization experiment in a single spot irradiated with a pulsed laser beam. The dark part is a transparent PET and the light ones are from light reflected by AgNPs.

There are a few stages in the laser treatment of AgNPs on PET substrate that could be clearly seen on Figure S2. At the very beginning (see 6 ms image), the circle-shaped heating wave spreads out while laser power is absorbed and scattered by metallic nanoparticles in the middle right part. Additional tests show that PET is fully transparent for the laser

wavelength used (more than 95%). As can be seen at 66 ms, we obtained a liquid phase of the substrate which means the AgNPs transfer sufficient heat to the substrate leading to the surface deformation and melting, that allows Ag NPs to diffuse inside the PET matrix.

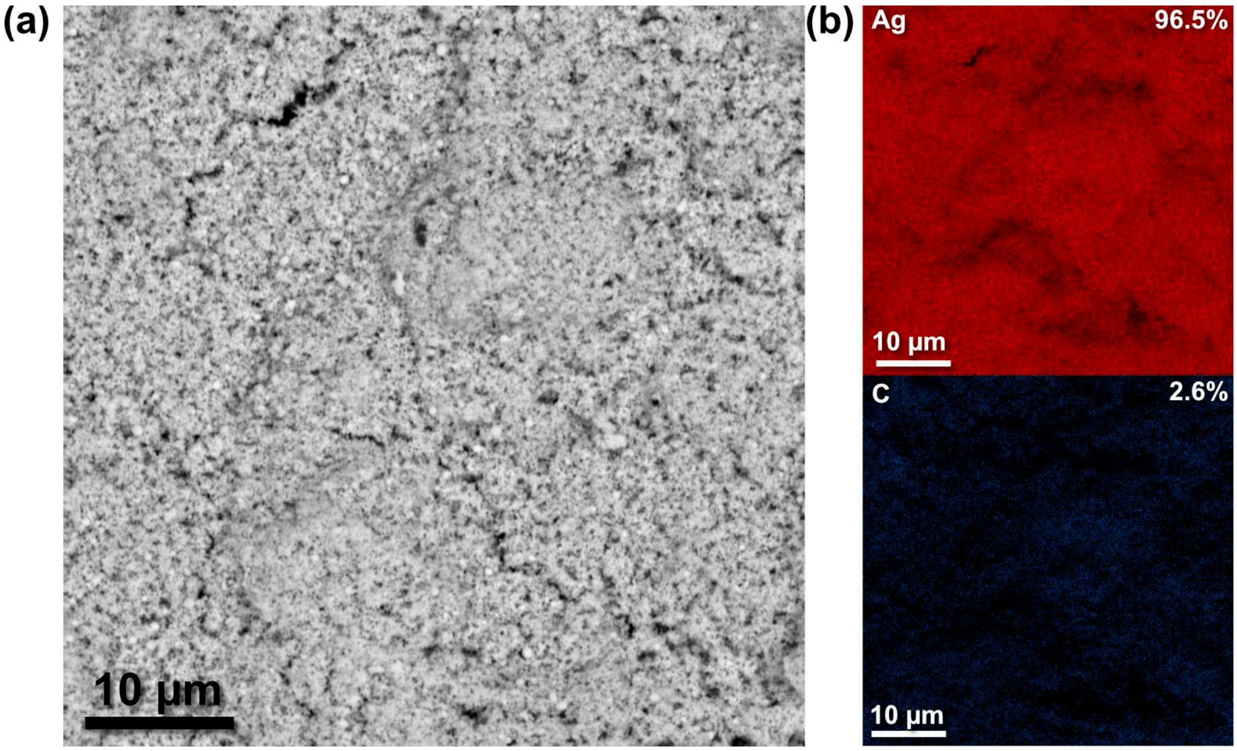

Figure S3 (a) Low magnification SEM image, and (b) Ag and C EDX maps of AgLIMPc.

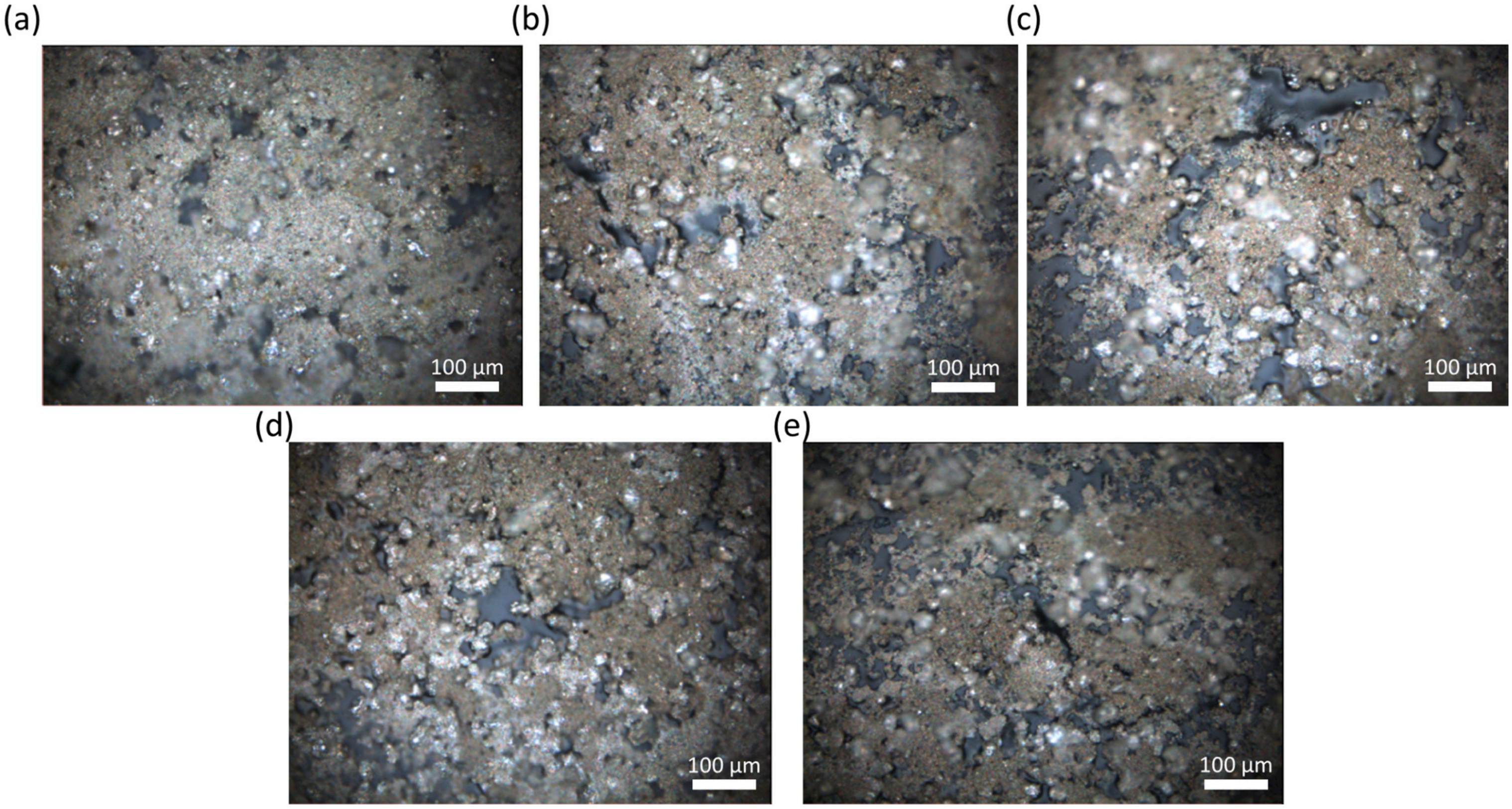

Figure S4 Optical images of AgLIMPc (a) before sonication; (b) after 1 minute; (c) after 2 minutes; (d) after 5 minutes and (e) after 10 minutes of sonication

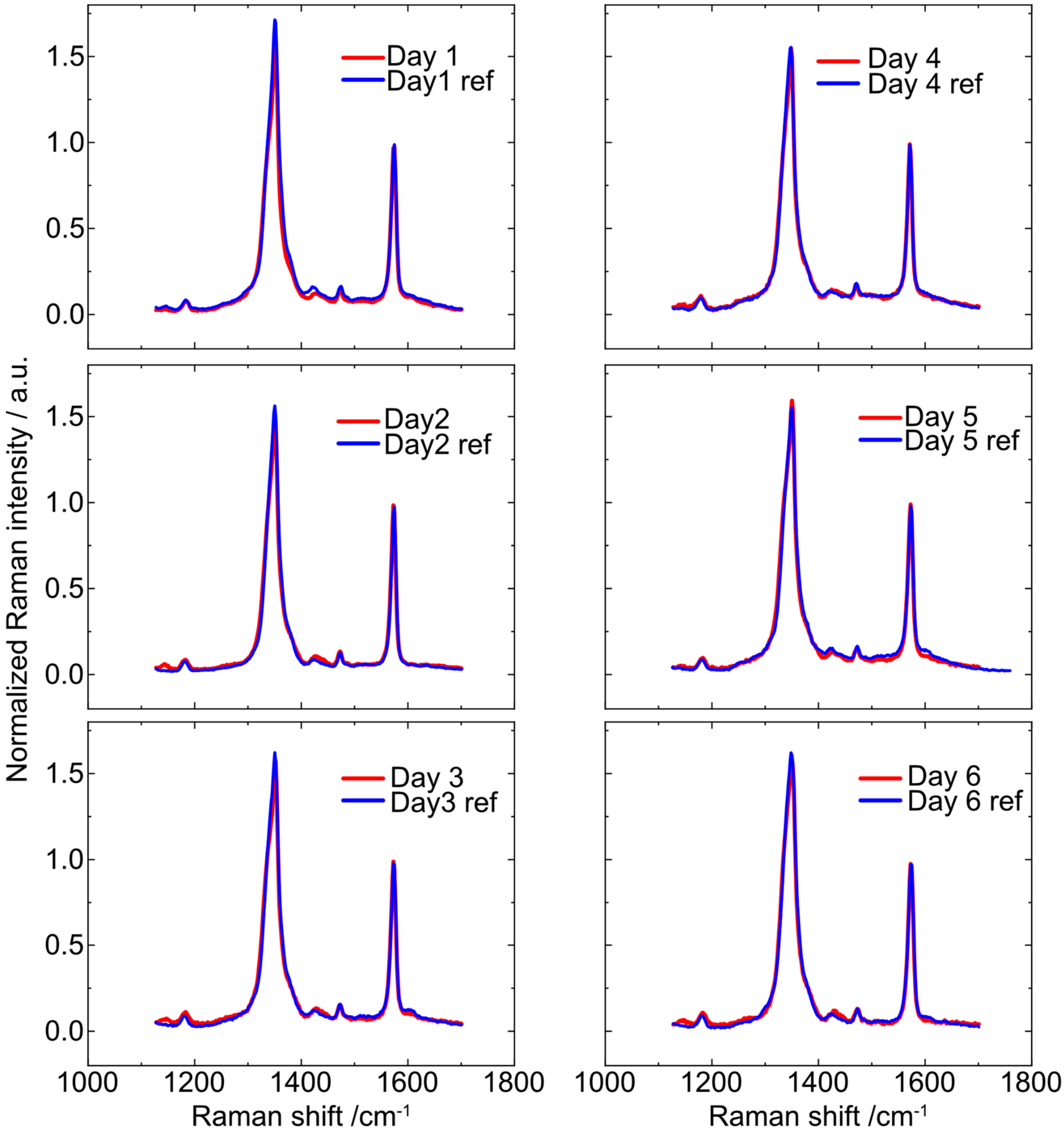

Figure S5 Raman spectra measured on the days 1 to 6 with the reference measured in water.

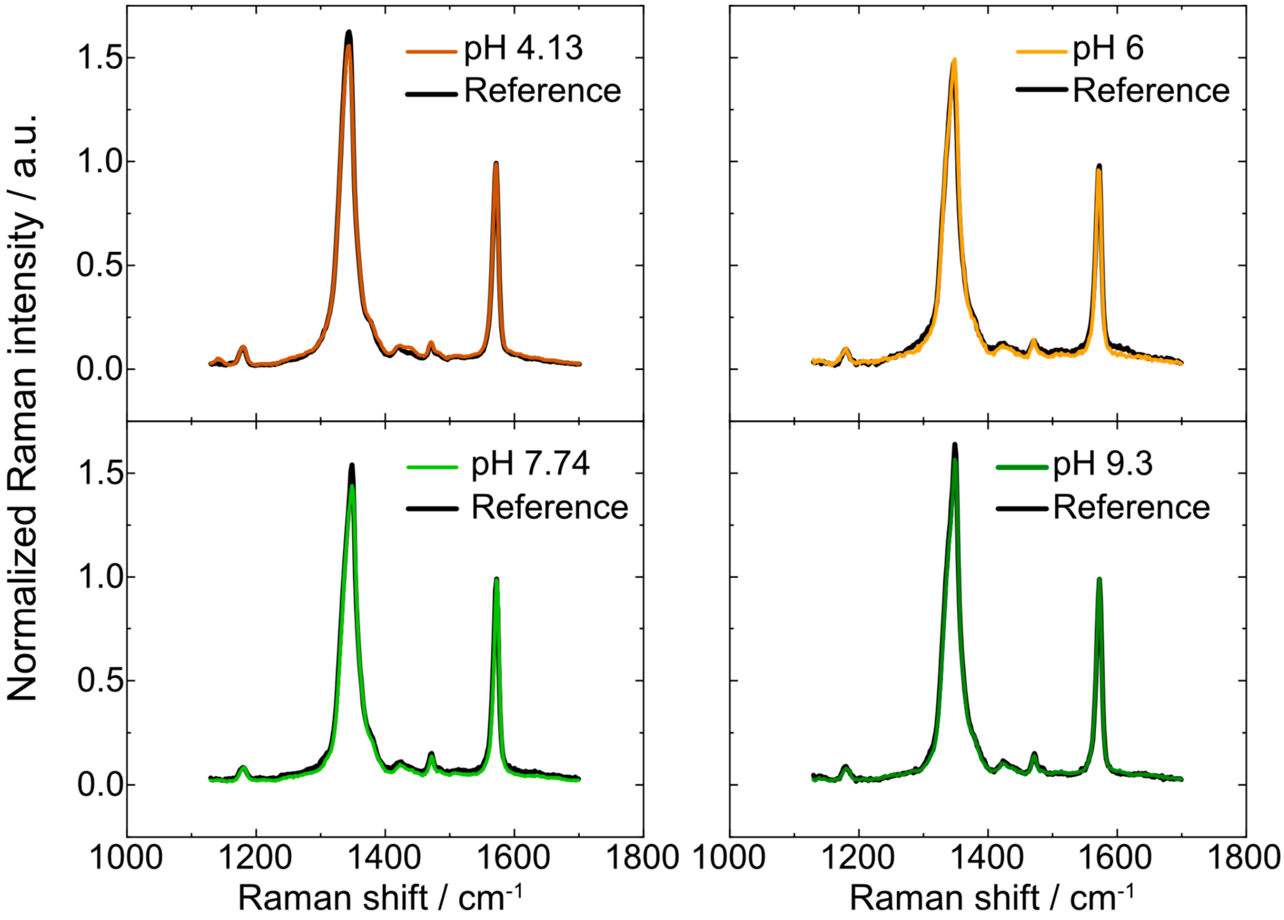

Figure S6 Raman spectra measured in the Britton-Robinson buffer with different pH.

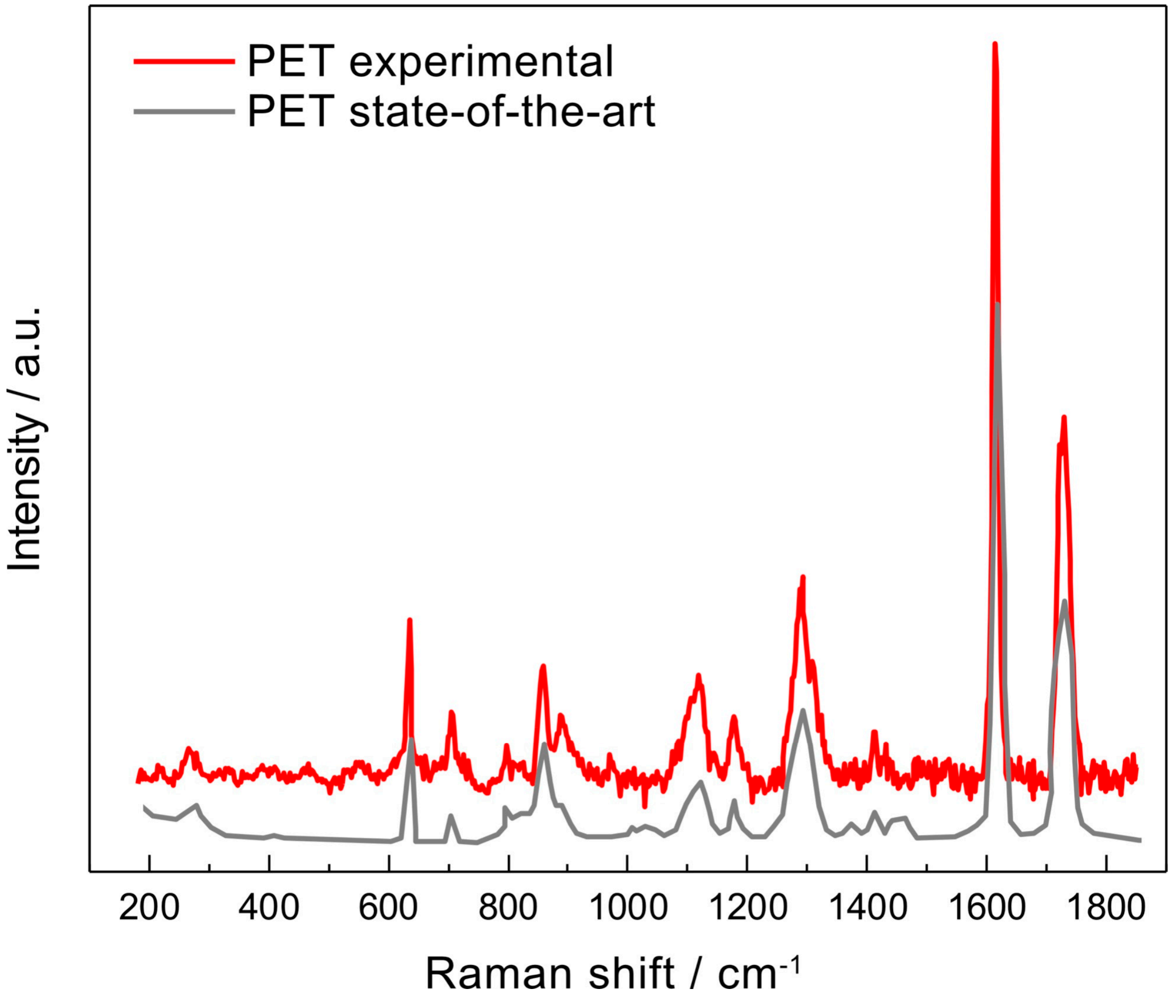

Figure S7 Raman spectra of PET. Red spectrum corresponds to experimental data, gray spectrum is taken from an open database (publicspectra)
